# Deep-sea hydrothermal vents as natural egg-case incubators at the Galapagos Rift

**DOI:** 10.1038/s41598-018-20046-4

**Published:** 2018-02-08

**Authors:** Pelayo Salinas-de-León, Brennan Phillips, David Ebert, Mahmood Shivji, Florencia Cerutti-Pereyra, Cassandra Ruck, Charles R. Fisher, Leigh Marsh

**Affiliations:** 1 Charles Darwin Research Station, Av Charles Darwin s/n, Puerto Ayora, Santa Cruz, Galapagos Islands Ecuador; 2 Galapagos Marine Research and Exploration (GMaRE), joint CDF-ESPOL research program, Charles Darwin Research Station, Santa Cruz, Galapagos Islands Ecuador; 3 Pristine Seas, National Geographic Society, Washington, D. C. USA; 4 Harvard Microrobotics Laboratory, Harvard University, Cambridge, MA USA; 5 Department of Ocean Engineering, University of Rhode Island, Narragansett, RI 02882 USA; 6 Pacific Shark Research Center, Moss Landing Marine Laboratories, 8272 Moss Landing Rd, Moss, CA 95039 USA; 7 Department of Ichthyology, California Academy of Sciences, 55 Music Concourse Drive, San Francisco, CA 94118 USA; 8 South African Institute for Aquatic Biodiversity, Private Bag 1015, Grahamstown, 6140 South Africa; 9 Save Our Seas Shark Research Center and Guy Harvey Research Institute, Nova Southeastern University, 8000 N Ocean Drive, Dania Beach, FL 33004 USA; 10 Department of Biology, Pennsylvania State University, University Park, State College, PA 16802 USA; 11 Ocean and Earth Science, University of Southampton, Waterfront Campus, Southampton, SO14 3ZH UK; 12 Marine Geoscience, National Oceanography Centre, European Way, Southampton, SO14 3ZH UK UK

## Abstract

The discovery of deep-sea hydrothermal vents in 1977 challenged our views of ecosystem functioning and yet, the research conducted at these extreme and logistically challenging environments still continues to reveal unique biological processes. Here, we report for the first time, a unique behavior where the deep-sea skate, *Bathyraja spinosissima*, appears to be actively using the elevated temperature of a hydrothermal vent environment to naturally “incubate” developing egg-cases. We hypothesize that this behavior is directly targeted to accelerate embryo development time given that deep-sea skates have some of the longest egg incubation times reported for the animal kingdom. Similar egg incubating behavior, where eggs are incubated in volcanically heated nesting grounds, have been recorded in Cretaceous sauropod dinosaurs and the rare avian megapode. To our knowledge, this is the first time incubating behavior using a volcanic source is recorded for the marine environment.

## Introduction

Despite being the largest biome, the deep sea remains the least explored ecosystem on earth^1,2^. In 1977, the discovery of the first deep-sea ecosystems supported by hydrothermal vent fluid emissions at the Galapagos Rift, challenged our views of ecosystem functioning and fueled new hypotheses about how life on earth could have originated around these chemically reactive environments^3–5^. Forty years later, we now know that hydrothermal vent ecosystems exist in every ocean basin, supporting rich communities and unique biological processes^6–9^. Initially considered isolated patches of life within a barren deep-sea floor, we are beginning to appreciate that these ecosystems interact with the surrounding environment and influence global geochemical cycles^7,8^. Most hydrothermal vent sites remain unexplored, and our understanding of the ecology of these ecosystems in most parts of the world remains limited. Furthermore, some of these chemosynthesis based ecosystems are now under threat from human activities and are targeted for exploitation of their mineral resources^10,11^. Here, we report for the first time a unique behavior where the Pacific white skate *Bathyraja spinosissima*, one of the deepest living of all known skate species^12^, uses active hydrothermal vent fields as a natural incubator for their external egg-capsules. To the best of our knowledge and understanding, this is the first time this incubating behavior at an active hydrothermal vent field has been recorded for a species within the marine environment.

**Figure 1:**
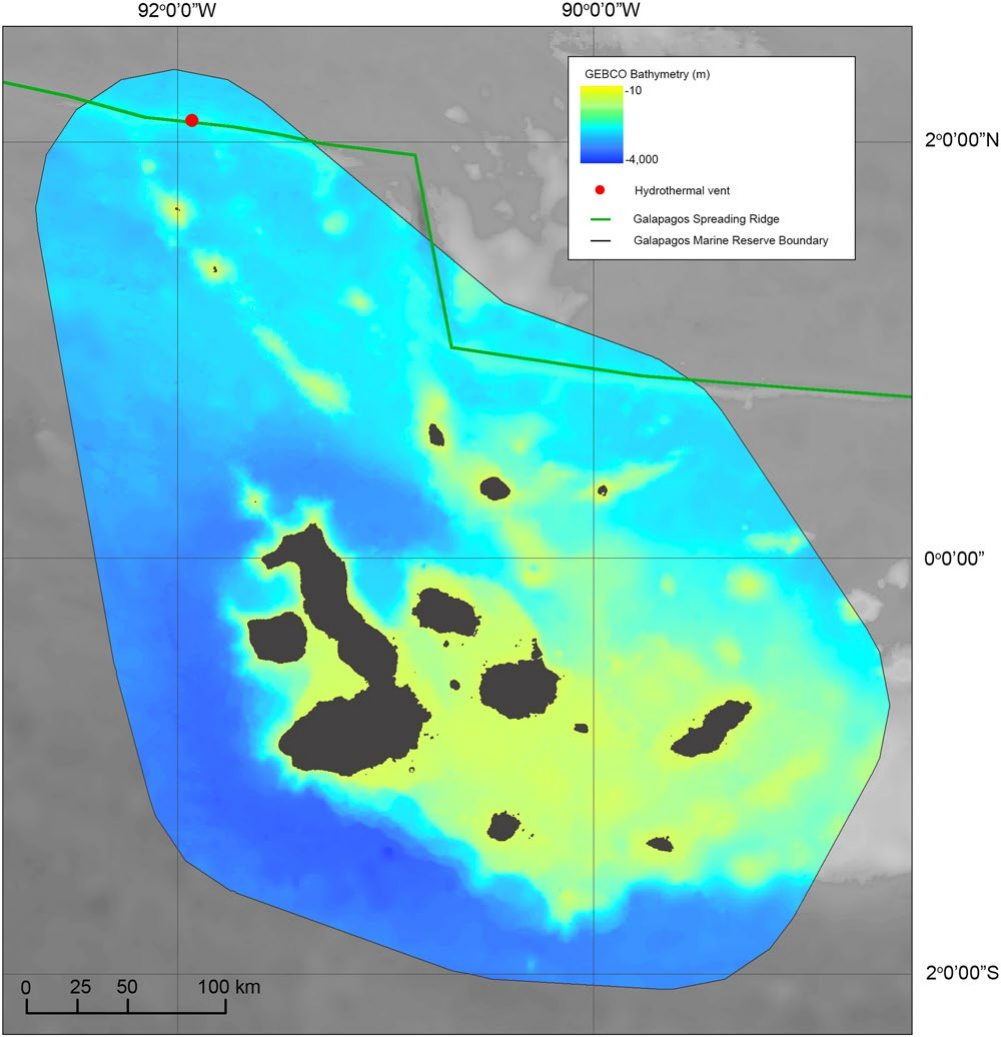
Bathymetric map of the Galapagos Marine Reserve (colored) with the location of the Galapagos Spreading Center (green line) and the Iguana-Pinguinos hydrothermal vent site (red dot) located 45 km north of Darwin Island. Gridded bathymetric data provided by the General Bathymetric Chart of the Oceans (GEBCO) 30 arc-second grid (accessed via http://www.gebco.net/). Map created in ESRI ArcMap (version 10.3.1).

The Galapagos Platform in the eastern tropical Pacific consists of 13 major volcanic islands and numerous seamounts that straddle the equator^13^. To the north of the archipelago and parallel to the equator, the Galapagos Spreading Center (GSC) extends for over 1000 km west to east, crossing the Galapagos Marine Reserve north of Darwin Island (Fig. 1). Previous exploration of the area revealed the presence of active hydrothermal vents, including the Iguanas-Pinguinos site that is located 45 km north of Darwin Islands^14^. This particular hydrothermal site was first described in 2008 as being in a mature/waning phase with macrofauna dominated by crabs, bivalves, anemones and shrimp^14^. The area is characterized by vigorous, active venting and dispersing clouds of ‘black-smoker’ hydrothermal plumes.

## Results

### Remotely Operated Vehicle (ROV) Surveys

**Figure 2:**
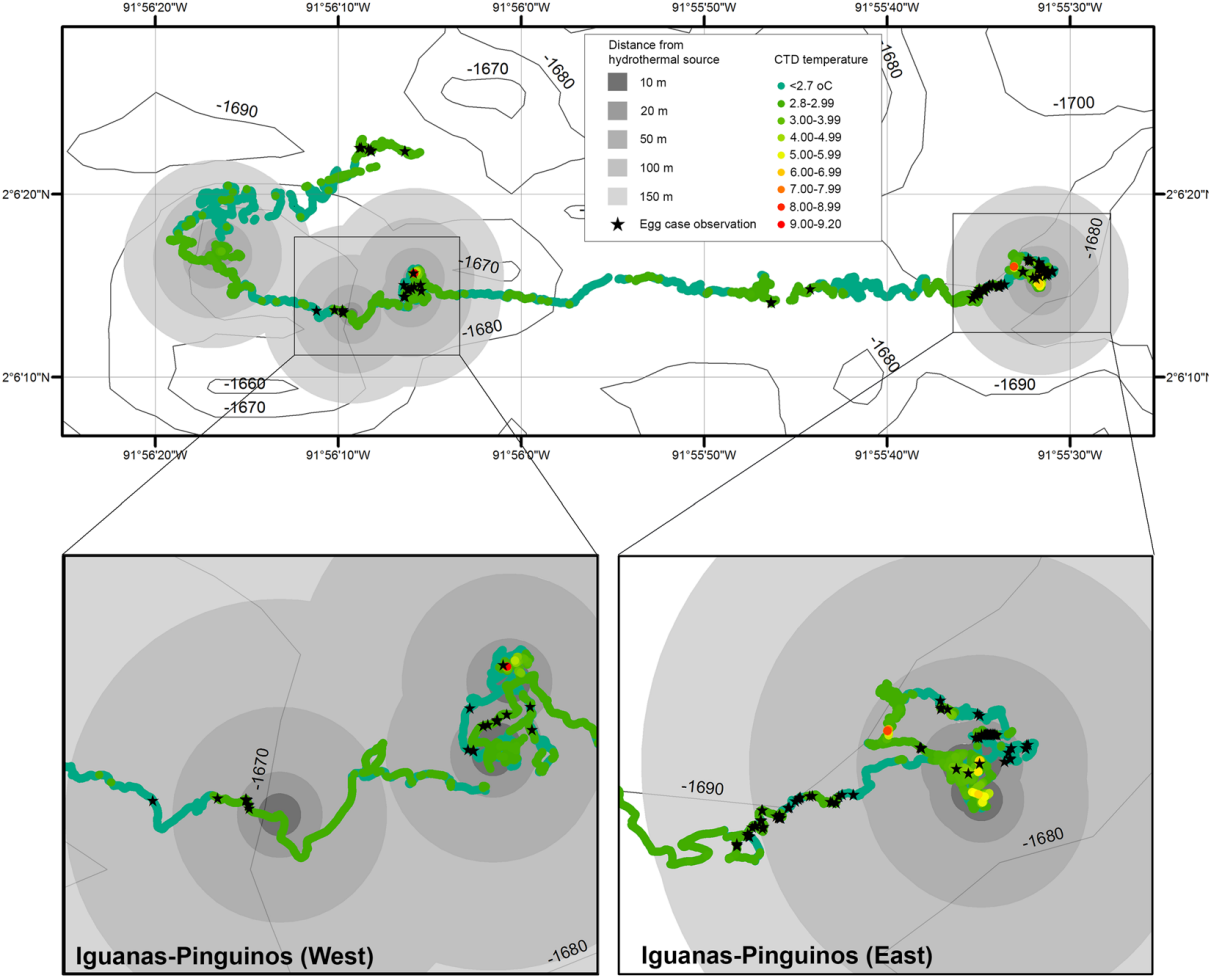
Higher resolution bathymetric map of the Iguanas-Pinguinos hydrothermal vent site at the Galapagos Marine Reserve. Map displays Hercules ROV CTD temperature (grouped in 1 °C increments) and position of individual egg-cases (black stars). Grey shaded areas denote distance from an active black smoker chimney. Contours generated from gridded bathymetric data provided by the General Bathymetric Chart of the Oceans (GEBCO) 30 arc-second grid (accessed via http://www.gebco.net/). Map and associated shapefiles created in ESRI ArcMap (version 10.3.1).

**Figure 3:**
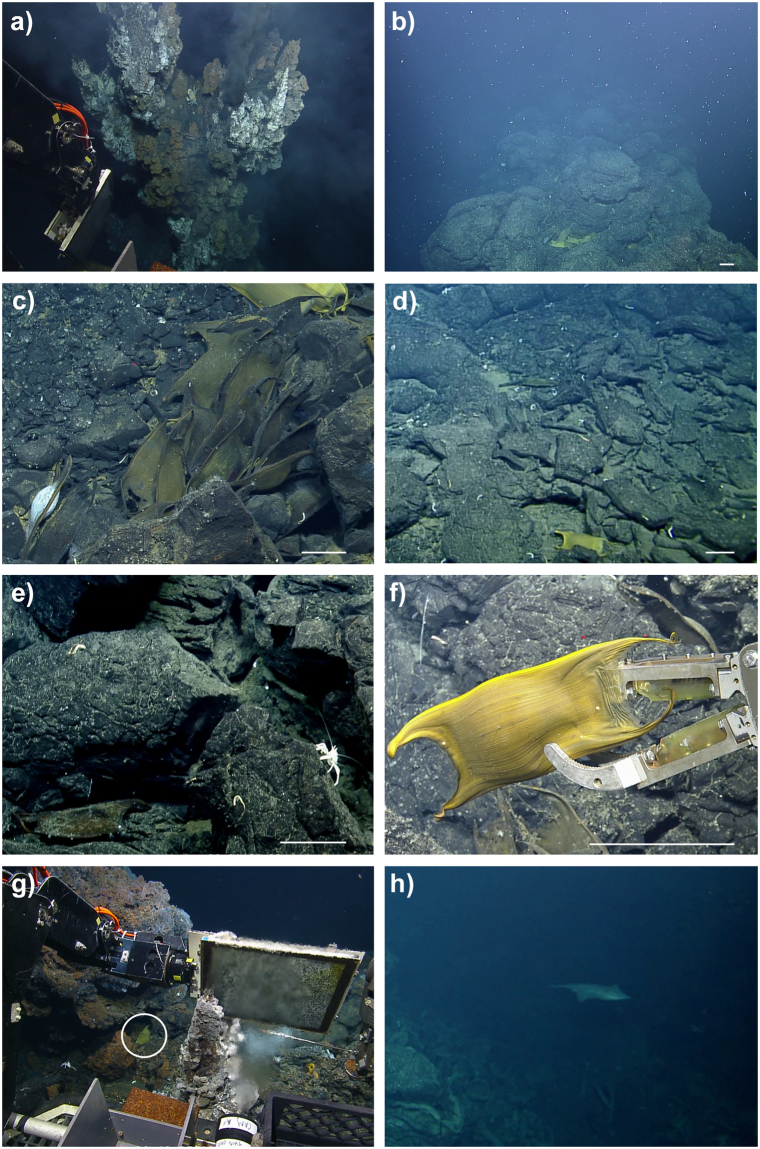
Hercules ROV imagery from the Iguanas-Pinguinos hydrothermal vent site at the Galapagos Marine Reserve (excluding H). All images were taken between 1666-1649 m depth. Scale bar represents 10 cm. (**a**) High-temperature black-smoker chimney at Iguanas-Pinguinos East; (**b**) egg-cases observed along ridge in close proximity to black-smoker; (**c**) clutch of egg-cases with dark brown coloration; (**d**) bright-yellow egg case among associated vent fauna; (**e**) older egg-case with signs of fouling; (**f**) collection of egg-case using the Hercules ROV robotic arm; (**g**) eggcase located within <1 m of active vent chimney (temperature recorded by the probe was 4.52 °C); (**h**) an adult *Bathyraja spinosissima* recorded on a previous dive at the Tempus Fugit hydrothermal vent site located about 750 km to the east, but also within the Galapagos Spreading Center. Footage and screenshots provided by Ocean Exploration Trust Inc (www.oceanexplorationtrust.org/).

Our surveys using the *Hercules* ROV recorded a total of 157 egg-cases in and around the Iguanas-Pinguinos vent field (Fig. 2; Supplementary material 1). Egg-cases were encountered right from the beginning of the dive when the ROV landed next to an active black smoker chimney located at 1660–1670 m depth (Fig. 3a). Over the duration of the 24-hour dive, most of the egg-cases seen were distributed within <150 m of two active black smoke chimneys (Figs 2, 3a). The largest deposition of egg-cases was on a basaltic ridge bathed in cloudy water venting from the nearby black smoker (Fig. 3b). The colors of live egg-cases ranged from golden to dark brown, suggesting that they were under different developmental stages (Fig. 3c–f). The majority of egg-cases visible lacked evidence of fouling suggesting they were recently deposited, however there were often older spent egg-case remnants under these, indicating that this deposition site has been used for many years (Fig. 3d). Some egg-cases were located within less than a meter from an active vent chimney (Fig. 3g).

### Environmental data

**Figure 4:**
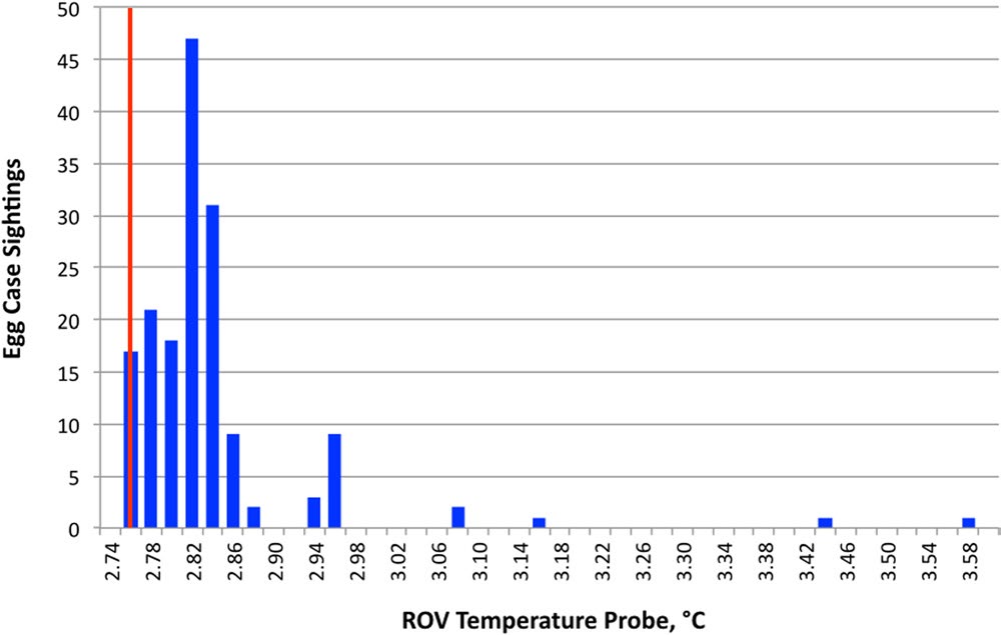
Number of egg case observations recorded as a function of water temperature detected by the *Hercules* ROV. The measured average background water temperature of 2.76 °C is indicated by the red vertical line. ROV altitude, reflecting the vertical position of the temperature probe, ranged 0.6–7.1 m. Recorded temperatures were consistently higher closer to the seafloor.

A temperature probe and a CTD sensor (Conductivity, Temperature, Depth) recorded temperatures during the duration of the dive. The temperature probe was located ~10 cm above the bottom of the ROV, while the CTD was located 1.5 m above the bottom of the ROV. Mean ambient bottom water temperature was 2.76 °C ±.0.01 SD and salinity was constant at 34.6 psu. Water temperature measured between 0.6–7.1 m above the seafloor was generally higher closer to the active smokers, but diffuse venting was also observed in some areas of the vent field along the survey track. The highest number of egg-cases (58%) in both areas of the Iguanas-Pinguinos vent field were recorded within <20 m of black smoker chimneys (Table 1). Anomalous temperatures exceeding 3.1 °C were occasionally recorded as high as 2.78 m above egg-cases in some places (Fig. 4). Over 89% of the egg-cases were observed while the ROV measured temperature above the ambient bottom water temperature of 2.76 °C.

**Table 1:** Number of egg-cases observed at different distances from the active Iguanas-Pinguinos hydrothermal sites.

Distance from active hydrothermal site	Number of egg-case observations		Total
	West	East	
10 m	3	4	7
20 m	29	55	84
50 m	2	14	16
100 m	1	30	31
150 m	0	10	10

### Species ID

We collected a total of 4 egg-cases using the *Hercules* ROV manipulator arm (Fig. 3f). During this collection, the ROV altitude was <1 m above the seafloor and the temperature probe measured 2.9 °C. Egg-cases were large, measuring 110 mm in length (excluding horns), and the surface was rough and striated^15^. The lateral keels were narrow with 10% of the maximum egg-case width. Horns were flattened and tapered towards the tips with tips becoming thin but not filamentous. Anterior horns where shorter and wider than posterior horns, the latter being 2 times larger than the length of the anterior horns (Fig. 3f).

Based on visual examination, the egg-cases resembled those of *Bathyraja spinosissima* (Beebe and Tee-Van, 1941) a species previously reported associated with hydrothermal vents in the Eastern Pacific and with a depth range that extends beyond 2900 m^15^. Although we did not encounter any adult skate specimens on our survey of the Iguanas-Pinguinos vent site, *B. spinosissima* were observed during a previous dive at the Tempus Fugit hydrothermal vent site located about 750 km to the east, within the Galapagos Spreading Center (Fig. 3h).

In addition to the visual examination of egg-cases and ROV video footage, analysis of 603 basepairs of the 5′ mitochondrial COI region (GenBank Accession no. MF158863) resulted in a 100% identity match to a sequence in the BOLD Systems database (GenBank Accession no. FJ164384). Although this specimen captured off Vancouver Island (Canada) was reported as *Bathyraja spinicauda*, the reported range for *B. spinicauda* is restricted to the North Atlantic and a taxonomic re-assessment of this specimen confirmed it to be *B. spinosissima*^15^. Therefore, we conclude that the egg-cases collected were *B. spinosissima*.

## Discussion

The presence of a *B. spinosissima* egg-case nursery (as described by^16^) in an active hydrothermal vent field, where even the temperatures several meters above the substrate were often higher than ambient water, implies that this species is utilizing heat at the active Iguanas-Pinguinos vent site to incubate its egg-cases. We hypothesize that this behavior is directly targeted to accelerate embryo development times. Deep-sea skates have some of the longest egg incubation times reported for the animal kingdom, with species of the same genera, such as *B. parmifera* in the Bering Sea, having incubation periods of 1290 days at water temperatures of 4.4 °C^17^. Assuming the ambient water temperature of about 2.76 °C is relatively constant year-round, and a development rate similar to *B. parmifera*, even a conservative correlation between temperature and embryonic development would suggest an incubation time of over 1500 days. This direct relationship between temperature and development time has been reported for several deep-sea organisms, including other oviparous Chondrichthyans^17,18^. While we recorded temperature increases of <1 °C above ambient in the water above where egg-cases were abundant, these are conservative measurements considering that the temperatures reported here were collected at an average altitude of 3.5 m above the seafloor. The temperatures directly on the seafloor, where diffuse venting and conductive heating may occur, are likely to be considerably higher.

Previous evidence of egg incubation at hydrothermal sites exists in the fossil record, where a group of neosauropod dinosaurs used soil thermo-radiance and moisture of hydrothermal origin to incubate their unusually larger eggs during the Cretaceous^19^. In contemporary times, megapode birds, such as the Polynesian megapode *Megapodius pritchardii*, burrow their nests in volcanically-heated soils on Niuafo’ou Island in Tonga^20^. Furthermore, several additional species of reptiles and birds actively seek specific soil temperatures to achieve optimal egg incubation. In the marine environment, aggregation of the egg-brooding deep-sea fish *Pychrolutes phrictus* and the cephalopod *Granelodone* spp. have been also recorded around “cold” seeps, where flows generate slight increases in temperature of 0.1–0.2 °C^21^. Despite these temperature anomalies, and the fact that *Granelodone boreopacifica* conducts the longest-known egg-brooding period of any animal (with over 1500 days of incubation time)^18^, egg occurrence was not correlated with temperature. This suggests that there may be other reasons for egg deposition in this area. For example, these cold seep sites could provide an additional food source that could influence the location of egg deposition^21^.

In addition to decreasing egg incubation periods, this is the first record of a hydrothermal vent habitat serving as an egg-case nursery site, a discrete habitat with extremely high densities of egg cases when compared to surrounding similar habitats^16^. Among the elasmobranchs, skates are the only group known to be strictly oviparous, where females produce large collagen egg cases containing a large yolk mass^22,23^. Egg-case nursery sites for members of the genus *Bathyraja* have been previously identified across most ocean basins and in diverse habitats like rocky reefs, submarine canyons, seamounts or even methane cold seeps^17,24,25^. Hydrothermal vent sites may also have other advantages as a juvenile nursery, although previous studies have revealed that juvenile skates of other species of the genus *Bathyraja* leave egg-case nurseries after hatching^16,26^.

One out of four species of Chondrichthyans are threatened with extinction, mainly as a result of over-fishing^27^. Deep-water Chondrichthyans species are among the least productive given their long turnover times (i.e. slower growth, later age at maturity and increased longevity) and, as a consequence, they may have higher extinction risk than other oceanic and continental shelf species^28,29^. Therefore, understanding their reproductive processes and key habitat requirements is vital to predict population stability and inform effective conservation strategies, especially under conditions of global change^17^. In March 2016, the Ecuadorian government created a 40,000 km^2^ marine sanctuary to protect unique underwater communities around Darwin and Wolf islands^30,31^. This fully non-extractive reserve also protects adjacent seamounts, and it includes the Iguanas-Pinguinos vent site, thus protecting the first known nursery for deep-water predators associated with active hydrothermal vents. In 2015, the North Pacific Fishery Management Council designated eight deep-sea skate egg-nurseries in the eastern Bering Sea as habitat areas of particular concern, and this represented the first official recognition of this habitat type worldwide^16^. Further research should focus on identifying and promoting the protection of additional Chondrichthyan deep-sea nurseries, given the continuous expansion of fisheries towards the deep-sea and the intrinsic vulnerability of this group of species^32–34^.

## Methods

### NA064 Galapagos platform cruise

In June 2015 we conducted a 10-day collaborative research cruise (NA064) aboard E/V *Nautilus* between the Ocean Exploration Trust, the Charles Darwin Foundation and the Galapagos National Park Directorate to explore deep-sea environment of the Galapagos Marine Reserve. On June 30^th^ 2015, we conducted dive H1439 to explore the active Iguanas-Pinguinos hydrothermal vent site (2° 6.322′ N, 91° 56.538′ W). All methods were carried out in accordance with relevant guidelines and regulations by the Galapagos National Park Directorate under research permits PC-26-15 & PC-45-15. All experimental protocols were reviewed and approved by a Galapagos National Park Directorate’s committee that asses animal care in research activities.

### ROV surveys

Exploration of the seafloor was carried out using the two-body Remotely Operated Vehicle (ROV) system *Argus* and *Hercules*, each rated for 4 km water depth. Video and still images of the sites were acquired using Insite Pacific Zeus Plus HD color video cameras on both vehicles, each equipped with a 10× mechanical zoom lens. CTD data was recorded using a calibrated Seabird FastCAT49 equipped with a circulating pump. The temperature probe used was an Omega PT100 RTD sensor housed in a custom titanium sheath (designed and fabricated by the Woods Hole Oceanographic Institution), which was calibrated against the Seabird FastCAT49 unit. These two units reported temperatures within 0.04 °C of each other. Seafloor navigation was performed using a combination of sensors including a LinkQuest Tracklink 5000 USBL system, RDI Workhorse Navigator 600 kHz DVL, IXSEA OCTANS 6000 fiber-optic gyrocompass, and a calibrated Paroscientific DigiQuartz depth sensor. DVLNAV software was used to process these data^35^.

### Sample collection and genetic analysis

Egg-case samples were collected using the ROV manipulator arm and placed on sample boxes aboard *Hercules* ROV for recovery. Once aboard the ship, two egg-cases were opened to sample for molecular analyses and further examination. Both egg-cases were at a very early development stage, with no clear presence of the embryo.

DNA was extracted from egg-cases with the QIAGEN DNeasy Blood & Tissue Kit (QIAGEN Inc., Valencia, CA). The 5′ region of the mitochondrial COI gene was sequenced on an AB 3130 genetic analyzer via the primer pair FF2d (5′-TTCTCCACCAACCACAARGAYATYGG-3′) and FR1d (5′-CACCTCAGGGTGTCCGAARAAYCARAA-3′) following the protocol outlined in^36^. Sequences were assembled in geneious 7.1.7^37^ and subsequently compared to species level barcode data in the BOLD Systems database (www.boldsystems.org).

## Electronic supplementary material

Supplementary Information
